# Performance of Commercially Available Rapid Serological Assays for the Detection of SARS-CoV-2 Antibodies

**DOI:** 10.3390/pathogens9121067

**Published:** 2020-12-19

**Authors:** Anwar M. Hashem, Rowa Y. Alhabbab, Abdullah Algaissi, Mohamed A. Alfaleh, Sharif Hala, Turki S. Abujamel, M-Zaki ElAssouli, Afrah A. AL-Somali, Fadwa S. Alofi, Asim A. Khogeer, Almohanad A. Alkayyal, Ahmad Bakur Mahmoud, Naif A. M. Almontashiri, Arnab Pain

**Affiliations:** 1 Vaccines and Immunotherapy Unit, King Fahd Medical Research Center, King Abdulaziz University, P.O. Box 80205, Jeddah 21859, Saudi Arabia; rymalhabbab@kau.edu.sa (R.Y.A.); maalfaleh@kau.edu.sa (M.A.A.); tabujamel@kau.edu.sa (T.S.A.); mzassouli@hotmail.com (M.-Z.E.); 2 Department of Medical Microbiology and Parasitology, Faculty of Medicine, King Abdulaziz University, P.O. Box 80205, Jeddah 21859, Saudi Arabia; 3 Department of Medical Laboratory Sciences, Faculty of Applied Medical Sciences, King Abdulaziz University, Jeddah 21859, Saudi Arabia; 4 Department of Medical Laboratories Technology, College of Applied Medical Sciences, Jazan University, Jazan 45142, Saudi Arabia; aalgaissi@jazanu.edu.sa; 5 Medical Research Center, Jazan University, Jazan 45142, Saudi Arabia; 6 Department of Pharmaceutics, Faculty of Pharmacy, King Abdulaziz University, Jeddah 21859, Saudi Arabia; 7 Pathogen Genomics Laboratory, Division of Biological and Environmental Sciences and Engineering (BESE), Thuwal 23955, Saudi Arabia; sharif.hala@kaust.edu.sa (S.H.); Arnab.Pain@kaust.edu.sa (A.P.); 8 King Abdullah International Medical Research Centre, King Saud University for Health Sciences, Ministry of National Guard Health Affairs, Jeddah 21859, Saudi Arabia; 9 Infectious Diseases Department, King Abdullah Medical Complex, Jeddah 21859, Saudi Arabia; afaalsomali@moh.gov.sa; 10 Infectious Diseases Department, King Fahad Hospital, Almadinah Almunwarah 11525, Saudi Arabia; fsalofi@moh.gov.sa; 11 Plan and Research Department, General Directorate of Health Affairs Makkah Region, Ministry of Health, Makkah 11176, Saudi Arabia; akhogeer@moh.gov.sa; 12 Department of Medical Laboratory Technology, University of Tabuk, Tabuk 71491, Saudi Arabia; aalkayyal@ut.edu.sa; 13 College of Applied Medical Sciences, Taibah University, Almadinah Almunwarah 42353, Saudi Arabia; abaMahmoud@taibahu.edu.sa (A.B.M.); nmontashri@taibahu.edu.sa (N.A.M.A.); 14 Center for Genetics and Inherited Diseases, Taibah University, Almadinah Almunwarah 42353, Saudi Arabia; 15 Research Center for Zoonosis Control, Hokkaido University, N20 W10 Kita-ku, Sapporo 001-0020, Japan; 16 Nuffield Division of Clinical Laboratory Sciences (NDCLS), University of Oxford, Oxford OX3 9DU, UK

**Keywords:** SARS-CoV-2, COVID-19, rapid assay, antibodies, serology

## Abstract

The coronavirus disease 2019 (COVID-19) pandemic, caused by the severe acute respiratory syndrome coronavirus 2 (SARS-CoV-2), continues to spread globally. Although several rapid commercial serological assays have been developed, little is known about their performance and accuracy in detecting SARS-CoV-2-specific antibodies in COVID-19 patient samples. Here, we have evaluated the performance of seven commercially available rapid lateral flow immunoassays (LFIA) obtained from different manufacturers, and compared them to in-house developed and validated ELISA assays for the detection of SARS-CoV-2-specific IgM and IgG antibodies in RT-PCR-confirmed COVID-19 patients. While all evaluated LFIA assays showed high specificity, our data showed a significant variation in sensitivity of these assays, which ranged from 0% to 54% for samples collected early during infection (3–7 days post symptoms onset) and from 54% to 88% for samples collected at later time points during infection (8–27 days post symptoms onset). Therefore, we recommend prior evaluation and validation of these assays before being routinely used to detect IgM and IgG in COVID-19 patients. Moreover, our findings suggest the use of LFIA assays in combination with other standard methods, and not as an alternative.

## 1. Introduction 

One reason behind the explosive spread of the severe acute respiratory syndrome coronavirus 2 (SARS-CoV-2) is the high rate of undocumented and asymptomatic cases which are able to spread the infection silently in the community [1]. Currently, real-time reverse transcriptase polymerase chain reaction (RT-PCR) is used as a standard method for SARS-CoV-2 diagnosis [2]. However, RT-PCR tests have many limitations including long turnaround time (~8–10 h), high cost, and the need for specified machines and highly skilled personnel. Furthermore, there has been a growing concern recently because of a global shortage in the supply of RNA extraction kits required for the RT-PCR assays. Moreover, RT-PCR can provide false negative results due to several reasons such as the timing as well as the quality of the collected swab samples, especially that the viral load declines in the upper respiratory tract with time [3,4]. Additionally, RT-PCR is only valid for patients with active shedding of infectious viruses or viral RNA and does not provide any data on the patients’ immune status. Therefore, there is an urgent need to complement such assays with a rapid test to quickly identify new, asymptomatic and recovered COVID-19 cases to aid in limiting SARS-CoV-2 spread.

Several companies have developed and produced rapid and specific immunoassays such as enzyme-linked immunosorbent assay (ELISA) and lateral flow immunoassays (LFIAs) to detect both IgM and IgG antibodies. Unlike ELISA, which is usually performed by well-trained personal in clinical laboratory settings, LFIAs overcome these challenges and provide a rapid, qualitative and simple point-of-care test (POCT) to detect the presences of both IgM and IgG antibodies. Prior to their use to screen and identify infected or immune individuals including asymptomatic as well as recovered patients, the performance of these assays must be evaluated and validated. Thus, in this study, we aimed to evaluate the performance of several commercially available immunoassays and compare them to pseudovirus microneutralization assay and our in-house developed and validated ELISA using samples derived from RT-PCR-confirmed COVID-19 patients and healthy controls.

## 2. Material and Methods

### 2.1. Samples and Testing Protocols

A total of 46 human serum samples from acute RT-PCR-confirmed COVID-19 patients collected at various time points ranging from day 3 to 27 from symptoms onset, and 15 serum samples from healthy subjects collected prior the COVID-19 pandemic were included in this study. All samples were examined for IgM and IgG responses by in-house ELISA and seven commercially available LFIAs, and for neutralizing antibodies (nAbs) by pseudovirus microneutralization assay. However, the total number of samples used to test the strips varied between commercial manufacturers due to variation in the number of supplied devices. Samples from healthy subjects were randomly selected from archived serum samples collected prior to the COVID-19 pandemic. All samples were anonymized and used based on ethical approvals obtained from the Unit of Biomedical Ethics in King Abdulaziz University Hospital (Reference No 245-20), the Institutional Review Board at the Ministry of Health, Saudi Arabia (IRB Numbers: H-02-K-076-0320-279 and H-02-K-076-0420-285), and the Global Center for Mass Gatherings Medicine (GCMGM) (No. 20/03A).

### 2.2. ELISA for Detection of SARS-CoV-2 Specific IgG and IgM Antibodies

In-house indirect ELISA based on the nucleocapsid (N) protein was performed as previously described [5]. Briefly, 96-well ELISA microplates were coated with in-house produced recombinant SARS-CoV-2 N protein at a concentration of 4 µg/mL in phosphate-buffered saline (PBS). After overnight incubation at 4 °C, the plates were washed three times with PBS containing 0.05% tween-20 (PBS-T), and blocked with 5% skim milk in PBS-T buffer at room temperature for 1 h. After blocking, plates were washed three times and serum samples at a dilution of 1:100 in PBS-T with 5% milk were added in duplicates and kept for 1 h at 37 °C. Next, plates were washed three times again with PBS-T and incubated with HRP-conjugated goat anti-human IgM or IgG antibodies (Jackson ImmunoResearch, West Grove, PA, USA) for 1 h. The plates were washed again, and incubated with TMB substrate (KPL, Gaithersburg, MD, USA) at room temperature for 30 min before terminating the reaction by adding 100 μL per well of stop solution (0.16 M sulfuric acid). The absorbance was measured at 450 nm using the ELx808™ Absorbance Microplate Reader (BioTek, Winooski, VT, USA). Positive samples were identified based on predetermined cut-off values (0.55 for IgM and 0.4 for IgG) as previously described [5].

### 2.3. Pseudovirus Neutralization Assay

The rVSV-ΔG/SARS-2-S*-luciferase pseudovirus was produced and titrated as previously described [6] using BHK21/WI-2 cells transfected with pcDNA expressing codon-optimized full-length SARS-CoV-2 S protein (GenBank accession number: MN908947) and infected with rVSV-ΔG/G*-luciferase (Kerafast, EH1020-PM). After 24-h incubation at 37 °C in a 5% CO_2_ humidified incubator, the supernatant containing the generated pseudovirus was harvested and titrated by measuring luciferase activity on Vero E6 cells. The titer was expressed as a relative luciferase unit (RLU). The microneutralization assay was then performed as previously described [6]. Briefly, heat-inactivated serum samples diluted at 1:20 in DMEM containing 5% FBS were mixed with diluted pseudovirus (equivalent to 2 × 10^4^ RLU) and incubated at 37 °C, in 5% CO_2_ for 1 h in duplicates. Then, 100 μL of the pseudovirus–serum mixtures were transferred onto confluent Vero E6 cell monolayers and incubated at 37 °C in a 5% CO_2_ humidified incubator for 24 h. After that, cells were lysed, and luciferase activity was measured using the luciferase assay system (Promega) according to the manufacturer’s instructions. Samples with ≥50% inhibition of luciferase activity compared to pseudovirus only control (no serum) were considered positive for nAbs against SARS-CoV-2.

### 2.4. SARS-CoV-2 IgG and IgM Antibodies by LFIA 

Seven available LFIA devices developed to detect SARS-CoV-2 IgM and IgG antibodies were tested in this study. The LFIA IgM/IgG rapid test manufacturers evaluated in this study included Qingdao Hightop Biotech Co. Ltd. (Qingdao, Shandong, China), BIOZEK medical (Apeldoorn, The Netherlands), Genrui Biotech Inc. (Shenzhen, China), CTK Biotech Inc. (Poway, CA, USA), Zhuhai encode medical engineering Co. Ltd. (Zhuhai, China), Xiamen Wiz Biotech Co., Ltd. (Xiamen, China) and GenBody Inc. (Cheonan, Korea). The kits were tested according to the manufacturer instructions by generally adding 10–20 µL from the test serum samples into the sample well plus several drops from the associated buffer. After 10–15 min incubation at room temperature, the results were recorded as positive, negative or invalid. Positive results showed colored bands at both the control and the test lines, whereas negative results only provided a colored band at the control line. The invalid results showed no colored band at the control line.

### 2.5. Statistical Analysis

Comparisons between groups were performed using one-way ANOVA and a Fisher’s LSD or Kruskal–Wallis test, and for all the proportions binomial 95% confidence intervals (CI) were calculated. Analyses were performed using GraphPad Prism 8 software.

## 3. Results

### 3.1. SARS-coV-2 IgM and IgG Antibodies Detection by ELISA

**Figure 1:**
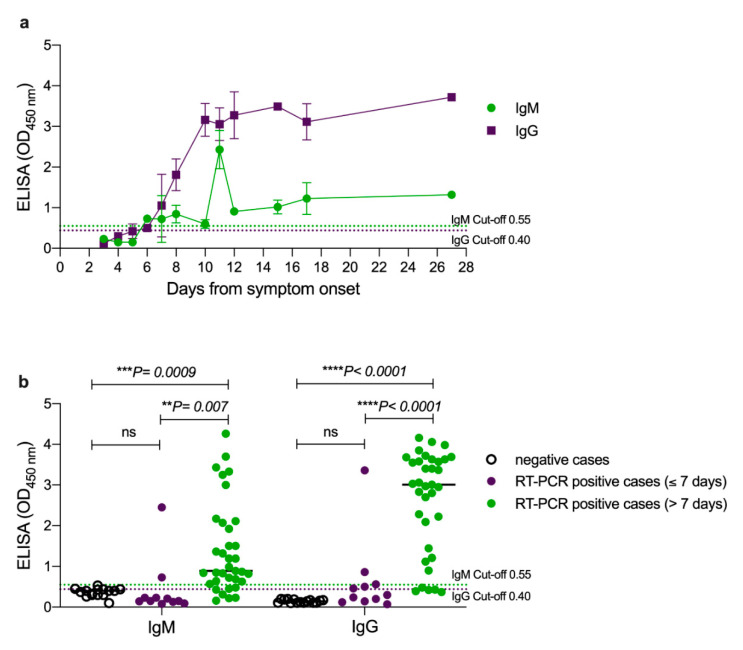
**SARS-CoV-2 IgM and IgG antibodies detection by ELISA.** A total of 46 serum samples collected from RT-PCR-confirmed COVID-19 patients were tested by ELISA to detect the levels of serum IgM and IgG antibodies specific for SARS-CoV-2 at several time points post symptom onset. Plots show ELISA OD reading for IgM and IgG antibodies (**a**) over time after symptoms onset (*n* = 46) and (**b**) for negative subjects (*n* = 15) as well as RT-PCR-positive cases before (*n* = 11) and after (*n* = 35) one week post symptom onset. The cut-off threshold values for IgM and IgG antibodies were 0.55 and 0.40, respectively. Each sample was tested in duplicates and error bar represents standard deviation. Statistics were calculated by one-way ANOVA and Fishers LSD test and *p* value < 0.05 was considered significant.

Here, we analyzed the relationship between the levels of serum antibodies and time since symptoms onset. A total of 46 samples were collected from RT-PCR-confirmed COVID-19 patients at several time points ranging from 3 to 27 days post symptoms onset, and the levels of SARS-CoV-2-specific IgM and IgG antibody responses were determined using in-house ELISA that we recently developed and validated [5]. As shown in Figure 1a, the level of IgM antibodies started to increase by the end of the first week and reached their highest levels at day 11 before dropping down to lower levels which were maintained above the initial levels until day 27. Similarly, IgG levels started to elevate by day 7 and peaked by day 10 to levels that remained high until day 27 post symptoms onset.

**Figure 2:**
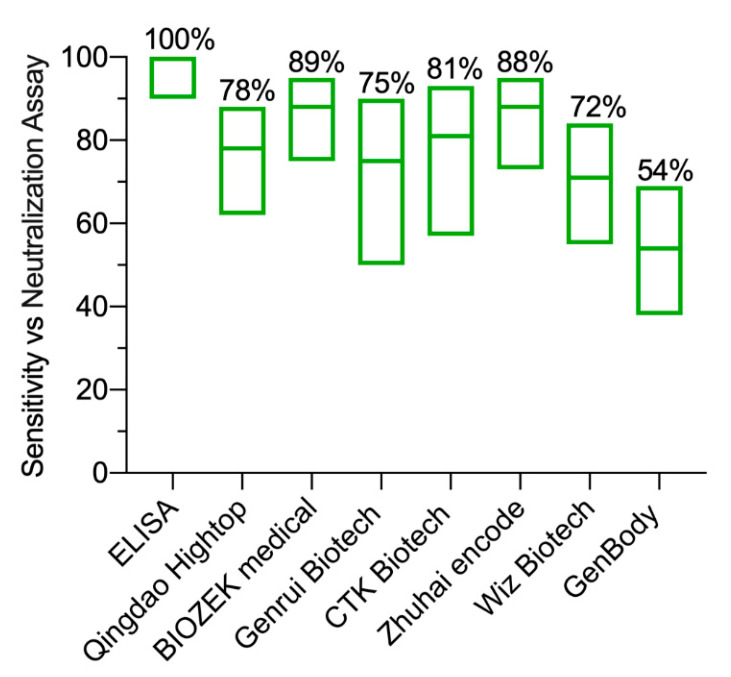
Sensitivity of SARS-CoV-2 antibodies detection by ELISA and lateral flow immunoassays (LFIA) compared to pseudovirus microneutralization assay. The results obtained from serum samples collected from RT-PCR-confirmed SARS-CoV-2 patients were used to calculate the sensitivity of the ELISA and LFIA assays compared to the pseudovirus assay. Floating plots show the sensitivity with 95% confidence intervals of the ELISA and the seven LFIA devices against the pseudovirus microneutralization assay. Means are shown as percentages on the top of each plot. Statistics were calculated by Wilsom/Brown methods.

The ELISA median optical density (OD) of the 15 negative samples was 0.4 (ranging from 0.03 to 0.53) for IgM and 0.14 (ranging from 0.1 to 0.2) for IgG. On the other hand, the median OD values of the 46 RT-PCR-confirmed positive cases were significantly higher for both IgM (0.82; ranging from 0.08 to 4.86) and IgG (2.75; ranging from 0.07 to 4.16). Based on the predetermined cut-off values [5], and the samples used in this study, the overall sensitivity of IgG ELISA vs RT-PCR was 83% (95% CI: 63–91%; 38/46). All eight false negative samples were from samples collected at early time points post symptoms onset six samples during the first week and two samples on day 8). Thus, the IgG positivity in samples collected at late time points post symptoms onset was detected in 33/35 of the RT-PCR-confirmed cases resulting in 94% sensitivity (95% CI: 81–99%). Importantly, all samples collected post day 8 were IgG positive, confirming our previous high sensitivity of IgG ELISA based on N protein [5]. Notably, the in-house IgG N-based ELISA showed 100% sensitivity compared to the pseudovirus microneutralization assay in which all the seropositive samples by the pseudovirus microneutralization assay were also positive by the IgG ELISA as shown in Figure 2. Importantly, the eight negative samples found by the IgG ELISA were also negative for nAbs. Furthermore, while two samples were found to be positive for IgG by ELISA but negative for nAbs, these two samples had low levels of anti-N IgG and undetectable levels of anti-spike protein antibodies (data not shown).

As expected, lower overall sensitivity of 75% was observed for the IgM ELISA (95% CI 83–63%; 30/46) compared to IgG ELISA, with 16 false negative samples divided between early (9 samples during the first week) and late time points (7 samples between days 8 and 11) post symptoms (Figure 1b). Nevertheless, all samples that were negative for IgG antibodies were IgM negative as well.

### 3.2. Detection of SARS-CoV-2-Specific Antibodies by ELISA and LFIA vs. RT-PCR

**Figure 3:**
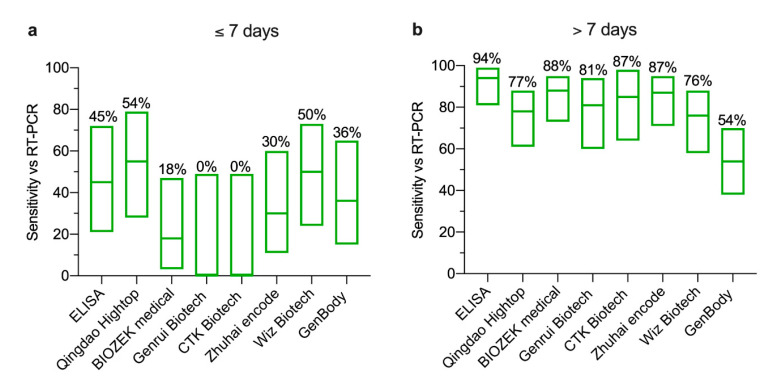
**Detection of SARS-CoV-2 antibodies by ELISA and LFIA compared to RT-PCR.** The results obtained from serum samples collected from a total of 46 RT-PCR-confirmed SARS-CoV-2 patients were divided into two groups based on the collection time points (one week and after one week post symptom onset). All groups including the negative control were tested for SARS-CoV-2 IgM and IgG antibodies by ELISA and seven LFIA devices. Floating plots show the overall sensitivity (**a**,**b**) with 95% confidence intervals of the ELISA and the seven LFIA devices against RT-PCR. Means are shown as percentages on the top of each plot from samples collected (**a**) during the first week and (**b**) after one week post symptom onset. Statistics were calculated by Wilsom/Brown methods.

Next, we compared the performance of seven LFIA devices as well as our in-house ELISA with RT-PCR results, considering the levels of serum antibodies at early and late time points post symptom onset (Figure 1), and the difference in measured targets by serological assays and RT-PCR. Therefore, any LFIA and ELISA positive results (IgG, IgM or both) were considered positive, and results were divided into two sets based on the peaking time points post symptoms onset (set 1: one week and set 2: after one week). To this end, the sensitivity of the seven tested LFIA devices and the ELISA compared to RT-PCR-positive cases for set 1 was very low ranging from 0% (95% CI 0–49%) to 54% (95% CI 28–79%) (Figure 3a). However, the sensitivity of the seven LFIA devices was increased to range from 54% (95%CI 38–69%) to 88% (95% CI 73–95%) for set 2 (Figure 3b). This increase was also observed with the ELISA achieving 94% (95% CI 81–99%) sensitivity (Figure 3b). Moreover, no false positives were detected from healthy control samples.

### 3.3. Detection of SARS-coV-2 Antibodies by ELISA vs. LFIA 

**Figure 4:**
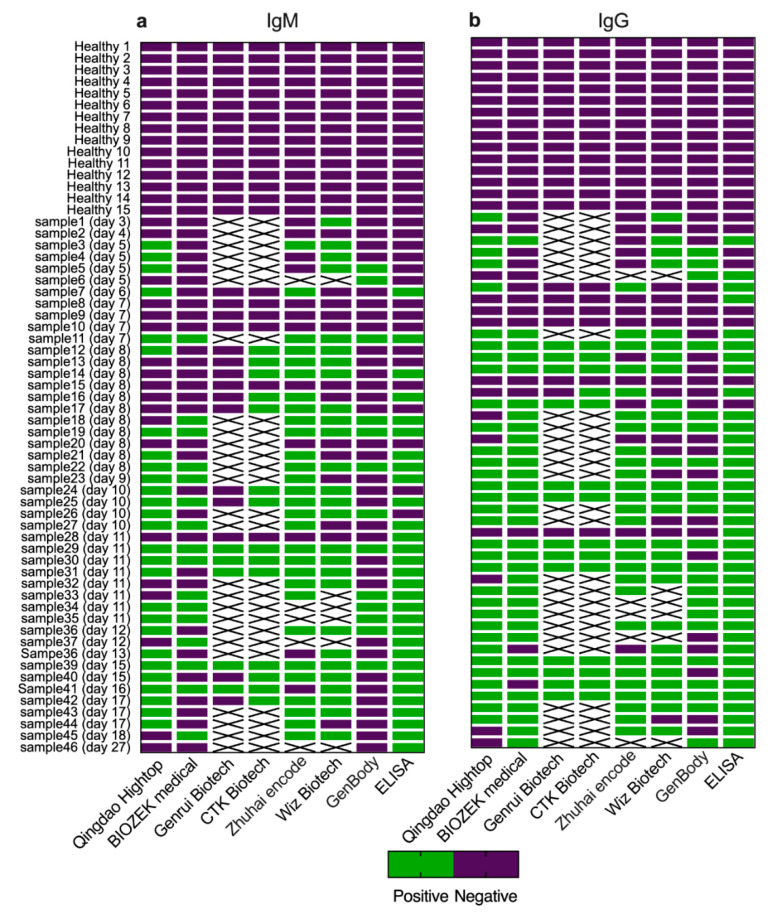
**Detection of SARS-CoV-2 antibodies by ELISA and LFIA**. Heat map of 46 serum samples of SARS-CoV-2 positive by RT-PCR and 15 plasma samples collected from healthy individual were tested by ELISA and seven LFIA devices for SARS-CoV-2 (**a**) IgM and (**b**) IgG antibodies.

**Figure 5:**
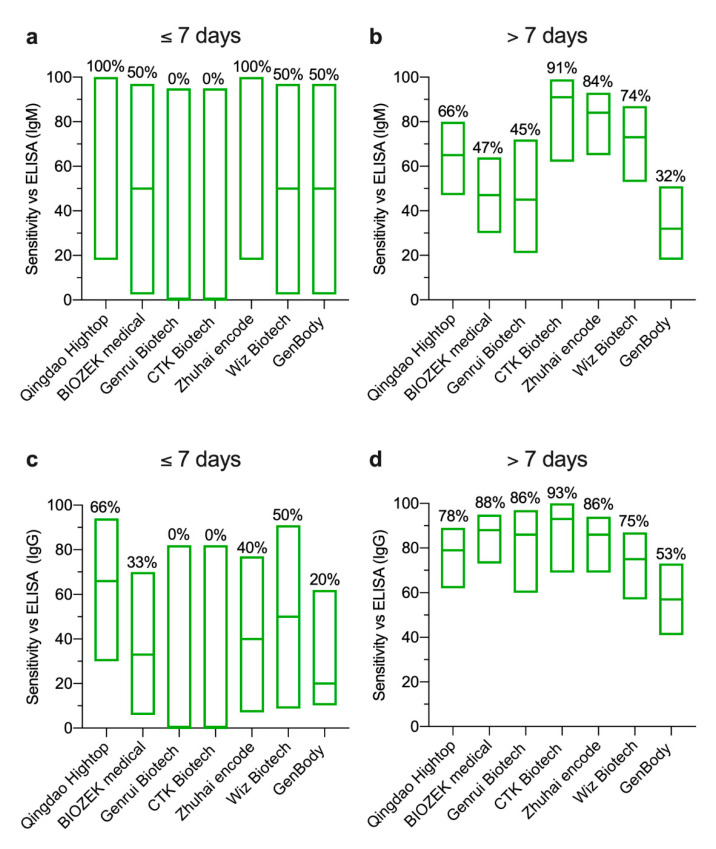
**Sensitivity of the LFIA devices compared to the in-house ELISA.** Floating plots show the sensitivity of (**a**,**b**) IgM and (**c**,**d**) IgG with 95% confidence intervals for each LFIA device against ELISA during the first week (**a**,**c**) and after one week post symptom onset (**b**,**d**). Means are shown as percentages on the top of each plot. Statistics were calculated by Wilsom/Brown methods.

Having demonstrated differences in the sensitivity between early and late time points post symptoms onset compared to RT-PCR, ELISA was used as an alternative standard assay to evaluate LFIA performance. However, since LFIA is qualitative and ELISA results are quantitative, any IgG or IgM OD reading that exceeded the ELISA cut-off value was considered positive as a qualitative measure of antibodies. Figure 4 summarizes the IgM and IgG antibodies results detected by the seven LFIA assays included in our study compared to the in-house ELISA. While no false positive results were observed out of the 15 healthy subjects consistent with the ELISA, several of the RT-PCR-confirmed cases that showed no antibody response by the in-house ELISA were found seropositive by many of the LFIA devices (Figure 4). As shown in Figure 5, IgM antibodies sensitivity ranged from 0% to 100% at early points (Figure 5a) and from 32% to 91% at late time points after symptoms onset (Figure 5b). IgG sensitivity of the seven LFIAs during the first week post symptoms onset ranged from 0% (95% CI 0–82%) to 66% (95% CI 30–94) (Figure 5c) and from 58% (95% CI 41–73%) to 93% (95% CI 68–99%) at late time points post symptoms onset (Figure 5d). The overall specificity for IgM and IgG detection by the seven LFIAs compared to our in-house ELISA ranged from 70% (95% CI 52–83%) to 100% (95% CI 85–100%) (data not shown).

## 4. Discussion 

LFIA is a rapid serological assay that provides cheap, easy and POCT to test and evaluate the level of exposure to SARS-CoV-2 in a given population. Although ELISA is cheap as well compared to RT-PCR, it cannot be used as a POCT and requires well-trained clinical laboratory staff and special tools and equipment to be performed. The determination of antibody responses to SARS-CoV-2 is crucial to identify immune individuals and therefore reduce anxiety, and can serve as a tool to release individuals from isolation or lockdown. Moreover, the fact that sampling time, particularly as infection progresses, could result in false negative results by RT-PCR, highlights the need to include serological testing in the testing protocol to improve the detection sensitivity. Furthermore, serological assays can also serve in quantifying and evaluating the immunogenicity of vaccines entering clinical trials. However, determining the sensitivity and specificity of such assays is important before releasing them for use in the clinical setting as high sensitivity and specificity are generally required in clinical diagnostics.

By utilizing cohort serum samples from RT-PCR-confirmed COVID-19 patients and healthy controls, we characterized the performance of commercially available LFIA assays obtained from seven different commercial manufacturers for the detection of SARS-CoV-2-specific IgM and IgG antibodies. While only 45% of the 11 RT-PCR-confirmed cases were detected by ELISA during the first week post symptoms onset, 94% of the 35 RT-PCR positive serum samples collected after one week from symptoms onset were ELISA seropositive. Of note, the in-house IgG N-based ELISA showed 100% sensitivity compared to the pseudovirus microneutralization assay confirming that not all samples from RT-PCR-confirmed cases seroconverted at the time of testing. On the other hand, the sensitivity of the tested LFIA showed high variability between manufacturers compared to either RT-PCR or ELISA, although their specificity seems to be high upon testing serum obtained from healthy subjects. Importantly, very low sensitivity and higher variability were seen during early time points post symptoms onset as expected, consistent with the often delayed seroconversion in COVID-19 patients which occurs around day 11 to day 19 post symptoms onset [7]. Similarly, we observed that antibody responses could peak around this time range, in which our ELISA results detected the initial increase of antibodies post day 7 of symptoms onset and peaking at day 11. Therefore, improving the sensitivity of these assays is crucial for early detection of antibodies post symptoms onset. 

The assessment in this study was based on a small number of samples and not powered to estimate of actual clinical performance of the LFIA devices or their agreement with RT-PCR and ELISA. Therefore, increasing the numbers and brands of the tested LFIA rapid assays would provide more assurance, however, the associated high cost might be considered as unjustified expense. Moreover, full assessment should also include different populations such as patients with different clinical presentations or immunological diseases, children and populations from different ethnicities and locations.

Collectively, rapid serological assays for SARS-CoV-2-specific antibody testing are important for diagnosis, contact tracing and epidemiological studies. Furthermore, such serological assays would be important to make informed decisions as some countries are considering relaxing some of their control measures such as lockdowns and travel restrictions. It also important to make sure these assays are accurate and have been appropriately validated. Although some of the tested LFIA assays in this study provide low sensitivity compared to ELISA and RT-PCR particularly at early time points after symptom onset, some of the devices were of high sensitivity. Importantly, our data demonstrated a high degree of variation in their sensitivity in detecting SARS-CoV-2-specific IgM and IgG antibodies and the need for proper validation of such assays before their deployments.
